# TNFα‐induced abnormal activation of TNFR/NF‐κB/FTH1 in endometrium is involved in the pathogenesis of early spontaneous abortion

**DOI:** 10.1111/jcmm.17308

**Published:** 2022-04-20

**Authors:** Yuting Wen, Meng Cheng, Lang Qin, Wenming Xu

**Affiliations:** ^1^ Department of Obstetrics/Gynecology Joint Laboratory of Reproductive Medicine (SCU‐CUHK) Key Laboratory of Obstetric Gynecologic and Pediatric Diseases and Birth Defects of Ministry of Education West China Second University Hospital Sichuan University Chengdu China; ^2^ Department of Obstetrics and Gynecology West China Second University Hospital Sichuan University Chengdu China; ^3^ The Reproductive Medical Center Department of Obstetrics and Gynecology West China Second University Hospital Sichuan University Chengdu China

**Keywords:** early spontaneous abortion, FTH1, inflammation, TNFα

## Abstract

Early spontaneous abortion (ESA) is one of the most common complications during pregnancy and the inflammation condition in uterine environment such as long‐term exposure to high TNFα plays an essential role in the aetiology. Ferritin heavy chain (FTH1) is considered to be closely associated with inflammation and very important in normal pregnancy, yet the underlying mechanism of how TNFα induced abortion and its relationship with FTH1 remain elusive. In this study, we found that TNFα and FTH1 were positively expressed in decidual stromal cells and increased significantly in the ESA group compared with the normal pregnancy group (NP group). Besides, TNFα expression was positively correlated with FTH1 expression. Furthermore, in vitro cell model demonstrated that high TNFα could induce the abnormal signals of TNFR/NF‐κB/FTH1 and activate apoptosis both in human endometrium stromal cells (hESCs) and in local decidual tissues. Taken together, the present findings suggest that the excessive apoptosis in response to TNFα‐induced upregulation of FTH1 may be responsible for the occurrence of ESA, and thus provide a possible therapeutic target for the treatment of ESA.

## 1 INTRODUCTION

Pregnancy is a physiological process that is often accompanied by a variety of complications, and it has significant effects on the physical and mental health in women of reproductive age. Spontaneous abortion is one of the most common pregnancy complications with a clinical incidence of around 15%, in which more than 80% cases occur before 12 weeks, also known as early spontaneous abortion (ESA).^1^, ^2^ Many factors may lead to the occurrence of ESA, including chromosomal abnormalities of the embryo, endocrine disorders of the mother, uterine dysplasia or malformation, immune dysfunction, and reproductive tract infection.^2^, ^3^, ^4^, ^5^, ^6^ However, about 50% of the aetiology and pathogenesis of the ESA are still elusive.

Researches have indicated that successful pregnancy requires the participation of inflammatory reaction, which is accompanied by the increase of cytokines and chemokines.^7^, ^8^, ^9^ Tumour necrosis factor‐alpha (TNFα) is thought to be one of the key cytokines and plays a vital role in the establishment and maintenance of pregnancy, whereas excessive TNFα has been linked to adverse pregnancy outcomes. Abnormally elevated levels of TNFα have been detected in both the local intrauterine and the serum of people and animals suffering abortion.^7^, ^10^, ^11^, ^12^ Previous studies have proposed using the TNFα expression as a diagnostic indicator of miscarriage and applying to guide its clinical treatment.^12^, ^13^ Although TNFα blocking agents have been applied for some chronic immune‐mediated inflammatory diseases during pregnancy, such as inflammatory bowel disease and psoriasis, and seem no harm for pregnancy and newborns, it is still controversial for its clinical safety.^14^, ^15^ Therefore, better understanding of the association between TNFα and ESA and its possible underlying mechanism will be helpful for avoiding the development of ESA.

Iron and iron‐binding proteins such as ferritin are also closely related to inflammation regulation during pregnancy. Ferritin is a ubiquitous and highly conserved protein that can bind to numerous irons and convert them into a nontoxic and bioavailable form to store inside its protein shell.^16^, ^17^ Abnormal alteration of ferritin content is usually linked with a variety of diseases, such as anaemia, pregnancy complications, kidney disease and Parkinson's disease.^18^, ^19^, ^20^, ^21^, ^22^, ^23^ It has been reported that ferritin concentration is higher in women with ESA than women with healthy pregnancy.^24^, ^25^ Besides, increased serum ferritin is also associated with higher rate of preterm birth.^21^, ^22^ As far as we know that mammalian ferritins are consist of two subunits named ferritin heavy chain (FTH1) and ferritin light chain (FTL), of them, FTH1 was considered to play an essential role in the process of pregnancy because its expression continues to increase throughout the gestation in response to progesterone.^26^ Besides, Ferreira, et al. delete the *Fth* gene in mice using homologous recombination and discover that the mice undergo early embryonic lethality at 9.5 gestation days.^16^ However, whether FTH1 fulfils functions in normal embryo implantation and the dysregulation of FTH1 poses implications on ESA remain unexplored.

Herein, we examined the changes of TNFα and FTH1 expression in patients suffered from ESA, and find that the expression of both TNFα and FTH1 were increased in local decidual tissues and show a strong correlation. In vitro experiments demonstrate that the activation of the TNFR/NF‐κB/FTH1 pathway induced by TNFα would lead to excessive apoptosis, and the activation was further confirmed in the patients; thus, the dysregulated pathway may represent a novel therapeutic target of ESA.

## 2 MATERIALS AND METHODS

### 2.1 Subject sample collection

Eighteen women with normal pregnancy who chose induced abortion were enrolled as NP group, and seventeen women suffered from early spontaneous abortion were enrolled as ESA group. Their decidual tissues were collected via artificial abortion‐vacuum aspiration. Ultrasound examination, menstrual cycle and blood hCG levels were used to confirm the viable intrauterine pregnancy and the gestational age of all subjects. All the subjects were enrolled according to the inclusion criteria as follows: i) The gestational age was ranged from 6 weeks to 12 weeks, ii) no malformation in reproductive tract and uterine, iii) no reproductive endocrine diseases or tract infections, and iv) no family history of heredity. This study was approved by the Ethical Review Board of West China Second University Hospital, Sichuan University. Informed consent was obtained from each subject in our study.

### 2.2 Cell culture

Human endometrium stromal cells (hESCs) were purchased from iCell Bioscience Inc (Shanghai, China) and cultured in DMEM/F12 medium (Gibco; Thermo Fisher Scientific, Inc.) supplemented with 10% fetal bovine serum (FBS; Gibco; Thermo Fisher Scientific, Inc.), 100 nM penicillin/streptomycin under an incubator with 5% CO_2_ and 37℃.

### 2.3 Real‐time PCR (RT‐PCR)

Total RNA was isolated from human decidual tissues or hESCs by the TRIzol^®^ reagent (Invitrogen; Thermo Fisher Scientific, Inc.) and was converted to cDNAs using PrimeScript RT Reagent (Takara, Japan) in accordance with the manufacturer's instruction. SYBR Green Fast qPCR Mix (Bimake, China) was used to conduct RT‐PCR on an iCycler RT‐PCR Detection System (Bio‐Rad Laboratories). Each assay was performed in triplicates for each sample, and *GAPDH* was applied as an internal control. The relative mRNA expression was quantified using the 2^−ΔΔCt^ method.^27^ The primers for RT‐PCR were listed in Table S1.

### 2.4 Western blotting

The proteins of human decidual tissues or the cultured cells were extracted and separated using 12.5% SDS‐polyacrylamide gels. Whereafter, the proteins were blotted into a polyvinylidene difluoride (PVDF) membrane (Millipore) and blocked with 5% skim milk for 1 h at room temperature. Subsequently, the membrane was incubated with primary antibodies, including anti‐GAPDH, anti‐TNFα and anti‐FTH1, for 4℃ overnight, and then incubated with corresponding secondary antibodies (goat anti‐mouse IgG or goat anti‐rabbit IgG, Abcam, US) for 1 h at room temperature. ECL Western blotting kit (Millipore) was applied to develop the blots.

### 2.5 Enzyme‐linked immunosorbent assay (ELISA)

The proteins were isolated from human decidual tissues, and the concentration of TNFα and FTH1 of the protein lysis buffers was detected by using Human TNF alpha Uncoated ELISA (Invitrogen, USA) and Human Ferritin Heavy chain 1 (FTH1) ELISA Kit (Mlbio, China). The procedure followed the instructions strictly. 3,3′,5,5′‐Tetramethylbenzidine (TMB) was applied to colour reaction. Absorbance was read at 450 nm after adding Stop Solution within 15 min. The concentration was calculated according to the standard curve.

### 2.6 Immunofluorescence staining (IF)

For the immunofluorescence staining of human decidual tissues, the deparaffinized and rehydrated slides were boiled in 10 mM citrate buffer (pH 6.0) to enable antigen retrieval. Then, the slides were blocked with 10% normal donkey serum for 1 h at room temperature and incubated with primary antibodies which included anti‐TNFα (Abclonal, China), anti‐FTH1 (Santa Cruz, USA), anti‐TNFR1 (Abcam, USA), anti‐TNFR2 (Proteintech, China), anti‐NF‐κB (Proteintech, China) and anti‐caspase3 (Abclonal, China) for 4℃ overnight. After that, the slides were washed by 1× PBS three times for 10 min each time, followed by 1‐hour room temperature incubation of corresponding fluorescent secondary antibodies (AlexaFluor 594 anti‐Rabbit, AlexaFluor 488 anti‐Mouse, Invitrogen, USA) and 4,6‐diamidino‐2‐phenylindole (DAPI). The slides were scanned and imaged by a digital slice scanner (Pannoramic MIDI, 3DHIESTECH, Hungary).

For the immunofluorescent staining of cultured cells, the slides were fixed with 4% paraformaldehyde and punched with 0.3% Triton X‐100 in PBS for 15 min. After that, the slides were blocked with 5% BSA and incubated with primary antibodies including anti‐NF‐κB (Proteintech, China). The following steps were consistent with the immunofluorescent staining of decidual tissues. The slides were observed and imaged by a laser scanning confocal microscope (Olympus, Japan).

### 2.7 Cell counting kit‐8 analysis

The proliferation and cell viability of hESCs were evaluated by cell counting kit‐8 (CCK‐8, Bimake, China) according to the manufacturer's instructions. 5000 cells were seeded in each well of a 96‐well plate and cultured overnight. After being treated with drugs, 10 μL CCK‐8 was added into each well, and the optical densities which reflected the cell numbers were determined at the absorbance of 450 nm after the incubation for 2 hours.

### 2.8 Apoptosis assays

After hESCs were treated with or without 10 ng/ml TNFα for 72 or 96 h, the cells were collected to stain with FITC‐conjugated Annexin V and propidium iodide (PI) using Annexin V‐FITC Apoptosis Detection Kit (BD Biosciences) in accordance with the recommendation of the manufacturer. The percentage of apoptotic cells was analysed by flow cytometry (BD Biosciences).

### 2.9 TUNEL assay

TUNEL assay was carried out in accordance with the guideline of TUNEL BrightGreen Apoptosis Detection Kit (Vazyme). After the sections of decidual tissues were carefully deparaffinized and rehydrated, the slides were permeabilizated with 20 μg/ml proteinase K, washed by 1 × PBS three times and balanced with 1 × Equilibration Buffer for 20 min. Then, BrightGreen Labeling Mix was applied to mark the apoptotic cells by incubating at 37℃ for 1 h. Subsequently, the slides were washed by 1 × PBS, stained with DAPI, and scanned and imaged by a digital slice scanner (Pannoramic MIDI, 3DHIESTECH, Hungary).

### 2.10 Cell cycle analysis

Detection of the cell cycle of hESCs treated with or without 10 ng/ml TNFα for 72 or 96 h follows the instructions of Cell Cycle Analysis Kit (4A Biotech). After being treated with or without TNFα, the cells were collected and fixed with 75% ethanol overnight. Then, PI was applied to stain the cells at 37℃ for 30 min. The determination of the cell cycle was conducted by Flow cytometry (BD Biosciences).

### 2.11 Statistical analysis

Statistical analysis was performed using SPSS 25.0; all the data were presented as means ± standard deviation (SD). For the analysis of two groups, a two‐tailed unpaired Student's t‑test was used; for the analysis of multiple comparations, one‐way ANOVA with Tukey's post hoc test was applied; for the analysis of the correlation between two groups, Person's correlation was conducted. All the in vitro experiments were repeated three times. The *p* values less than 0.05 were determined as significant difference.

## 3 RESULTS

### 3.1 Increased expression of TNFα and FTH1 were found in the ESA group

**FIGURE 1:**
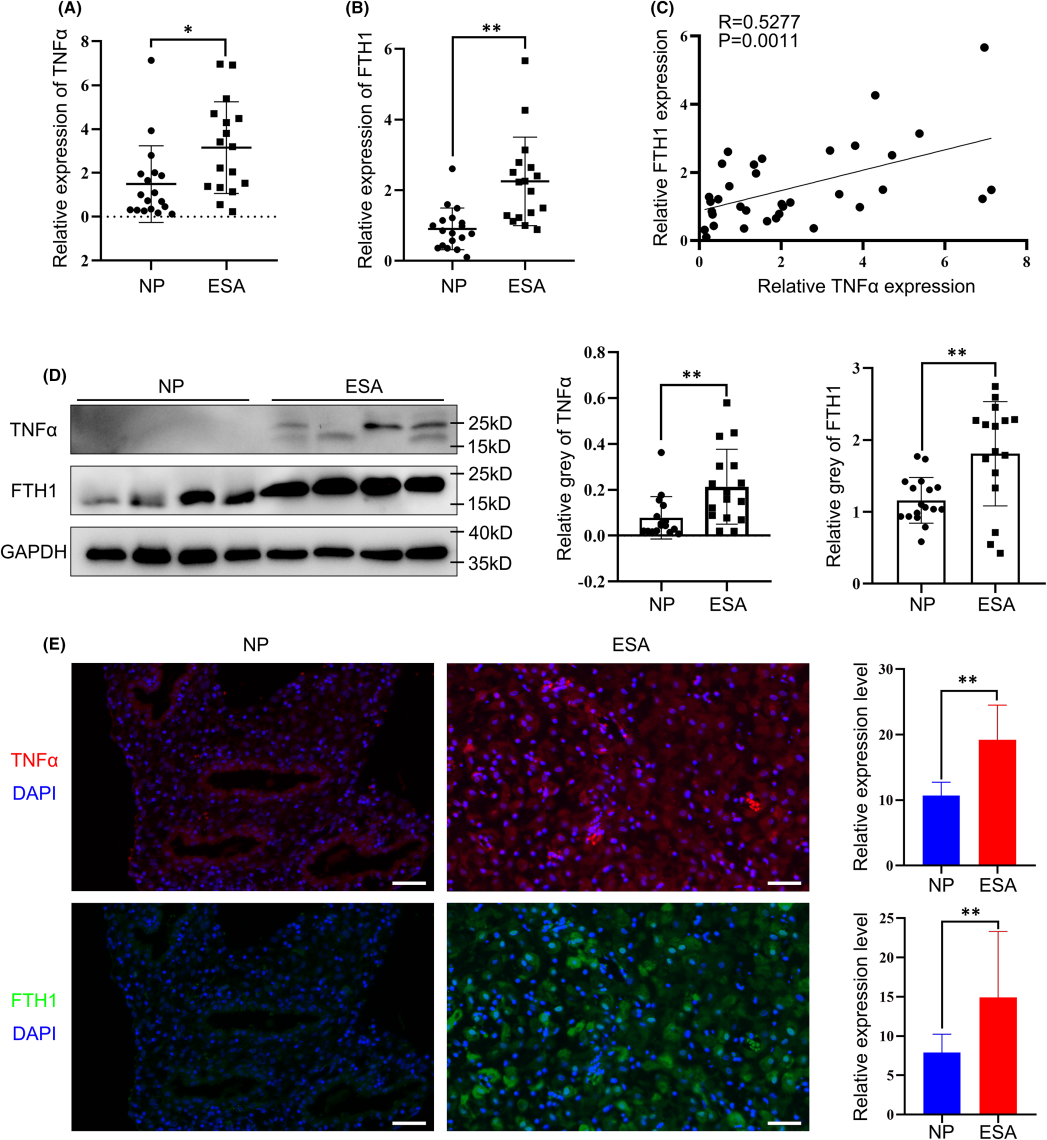
Expression of TNFα and FTH1 was upregulated in the ESA group compared with the NP group.(A and B) RT‐PCR results showed that the mRNA expression of TNFα (A) and FTH1 (B) was increased in the decidua from the ESA group (*n* = 17) compared with the NP (*n* = 18) group. NP, normal control; ESA, early spontaneous abortion. *p < 0.05, **p < 0.01. (C) Correlation analysis showed a significant positive correlation between the mRNA expression of TNFα and FTH1. (D) The protein expression of TNFα and FTH1 was increased in the decidua from the ESA group (*n* = 16) compared with the NP (*n* = 17) group by Western blotting. The middle and right figures were the statistical analysis of the normalized expression of TNFα and FTH1. **p < 0.01. (E) The immunofluorescence staining of the decidua from the NP group and ESA group showed that TNFα and FTH1 were positively expressed in decidual stromal cells, and manifested stronger intensities in the ESA group compared with the NP group. The statistical diagrams indicated total immunofluorescence staining intensities evaluated by ImageJ. NP, *n* = 4; ESA, *n* = 4. **p < 0.01. Scare bar = 50 μm

The clinical characteristics of patients from the NP group and the ESA group were investigated and no significant differences in age and BMI between the two groups were found (Table 1), suggesting the samples from the two groups could be used for next analysis. To understand the relationship between TNFα, FTH1 and ESA, RT‐PCR was performed to detect the mRNA expression levels of TNFα and FTH1 in decidual tissues from the two groups, and results showed significant increases of TNFα and FTH1 relative expression in the ESA group compared with the NP group (Figure 1A,B). Interestingly, further correlation analysis revealed a remarkably positive correlation between the relative mRNA expression of TNFα and FTH1 (*N* = 18 for the group of NP, *N* = 17 for the group of ESA; *p* = 0.0011) (Figure 1C). Consistently, Western blotting and ELISA displayed similar results, which further confirmed that the upregulation of TNFα and FTH1 in the ESA group and their positive correlation with each other (Figure 1D; Figure S1A‐C). Additionally, immunofluorescence staining of decidual tissues was applied to demonstrate that both TNFα and FTH1 were located and upregulated in the stromal cells (Figure 1E). Since FTH1 and FTL made up ferritin together, we further explored the expression of FTL in decidua tissues, and similarly discovered an obvious elevation of FTL in the ESA group and a positive correlation with relative TNFα expression (Figure S1D,E). Taken altogether, we speculated that the development of ESA may result from the unusual expression of TNFα and ferritin.

**TABLE 1:** Clinical characteristics of subjects

	NP	ESA	*p*
*N*	18	17	—
Age (year)	30.29 ± 3.34	30.56 ± 3.48	ns
BMI (kg/m^2^)	20.94 ± 2.44	21.06 ± 2.29	ns
Gestational age (week)	7.55 ± 1.20	9.66 ± 1.41	**

Note

All values were presented as means ±standard deviation. ns, no significance; **p < 0.01.

### 3.2 TNFα induced the apoptosis of human endometrium stromal cells

**FIGURE 2:**
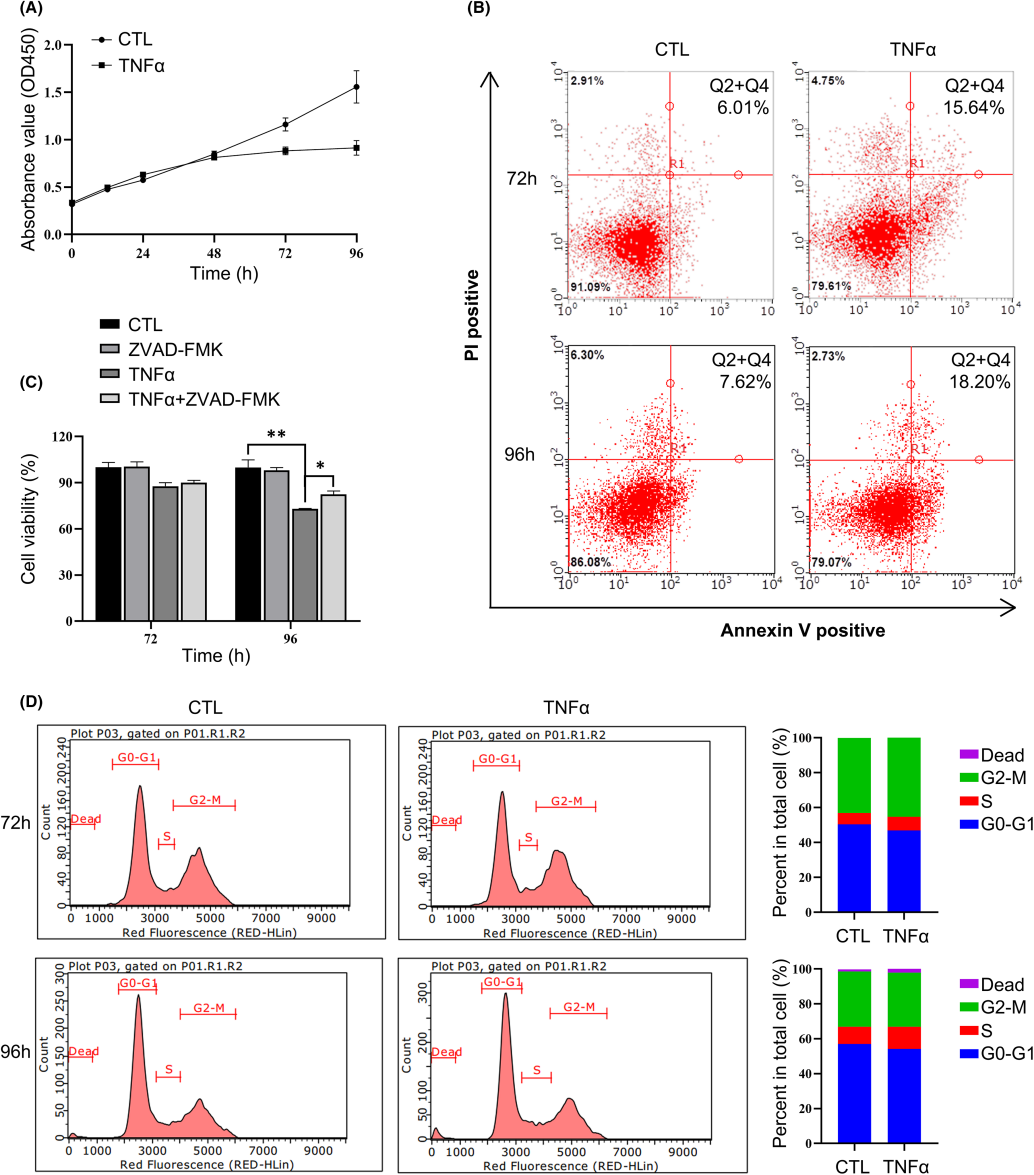
TNFα inhibited cell proliferation of stromal cells by inducing apoptosis but not altering the cell cycle. (A) Long‐time treatment of 10 ng/ml TNFα inhibited the proliferation of stromal cells. (B) Long‐time exposure of TNFα (10 ng/ml, 72 or 96 h) induced apoptosis of cells. (C) The partial rescue effect of pan‐caspase inhibitor ZVAD‐FMK on TNFα‐induced cell death in hESCs. Cells were exposed to TNFα (10 ng/ml, 72 or 96 h) with or without ZVAD‐FMK (10 μM). *p < 0.05, **p < 0.01. D, The cell cycle of the stromal cells was not affected by the treatment of TNFα (10 ng/ml, 72 or 96 h). The left pattern showed the cell cycle detected by flow cytometry, and the right pattern manifested the corresponding statistical graphs of cell proportion at different cycle stages

To further understand the effect of abnormal expression of TNFα in decidual stromal cells, 10 ng/ml TNFα was used to stimulate hESCs to imitate the intrauterine environment of patients with ESA. After being treated with TNFα for 72 h, the cell proliferation was inhibited and the inhibition was more obvious for 96 h (Figure 2A). Previous studies suggested that the inhibition of cell proliferation may be due to the increase of cell death and the arrest of cell cycles.^28^ Hence, the alteration of cell death and cell cycle of hESCs treated with or without TNFα for 72 or 96 h were analysed, and the percentage of apoptotic cells were found to be increased (Figure 2B), besides, it could be partially rescued by apoptosis inhibitor ZVAD‐FMK, but not ferroptosis inhibitor ferrostatin‐1 (Fer‐1) or necroptosis inhibitor necrostatin‐1 (Nec‐1) (Figure 2C; Figure S2A). While there was no effect on the cell cycle after being treated with TNFα (Figure 2D), these findings indicated that the inhibition of hESC cell proliferation induced by TNFα resulted from the increase of apoptosis rather than the arrest of cell cycle. Some studies have reported toxic effects of TNFα on trophoblast cells during pregnancy^29^; thus, we further investigated the impact of TNFα on the cell viability of human first‐trimester extravillous trophoblast cells (HTR8/SVneo); however, the HTR/SVneo showed a normal growth after long‐time treatment of TNFα (Figure S2B), indicating that the stromal cells were more likely to be affected by TNFα treatment.

### 3.3 The expression of FTH1 was upregulated by TNFα in hESCs

**FIGURE 3:**
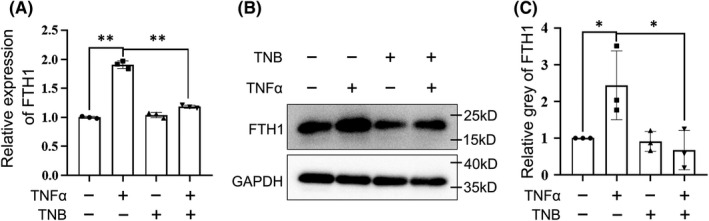
TNFα induced the increased expression of FTH1. (A and B) Cells were stimulated by TNFα (10 ng/ml) with or without TNB (2 μg/ml) for 48 h. Both RT‐PCR (A) and Western blotting (B) showed that the expression of FTH1 was upregulated by the treatment of TNFα, and could be neutralized by anti‐TNFα TNB. (C) Statistic analysis of the Western blotting. *p < 0.05, **p < 0.01

Given that the expression of FTH1 and FTL were significantly positively correlated with the expression of TNFα in the human decidual tissues, we therefore aim to examine whether TNFα could regulate FTL and FTH1 expression. As expected, the expression level of FTH1 was changed throughout processing time and rose to a peak after being stimulated with 10 ng/ml TNFα for 48 h (Figure S3A,B). However, 48 h treatment of TNFα did not induce the upregulation of FTL (Figure S3C). Subsequently, different concentrations of TNFα Nanobody (TNB) were used to attenuate the stimulation of TNFα, of which 2 μg/ml TNB showed complete neutralization (Figure S3D). Therefore, 2 μg/ml TNB was used to further verify the action of TNFα on FTH1. It was not surprising that the upregulation of FTH1 induced by TNFα was blocked by TNB (Figure 3), demonstrating that the expression of FTH1 was mediated by TNFα. Intriguingly, TNFα could not regulate FTH1 expression in HTR8/SVneo cells (Figure S3E), further implying the detrimental role of TNFα‐induced FTH1 in stromal cells, which closely associated with ESA.

### 3.4 Apoptosis was mediated by TNFα/TNFR/NF‐κB/FTH1 axis in hESCs

**FIGURE 4:**
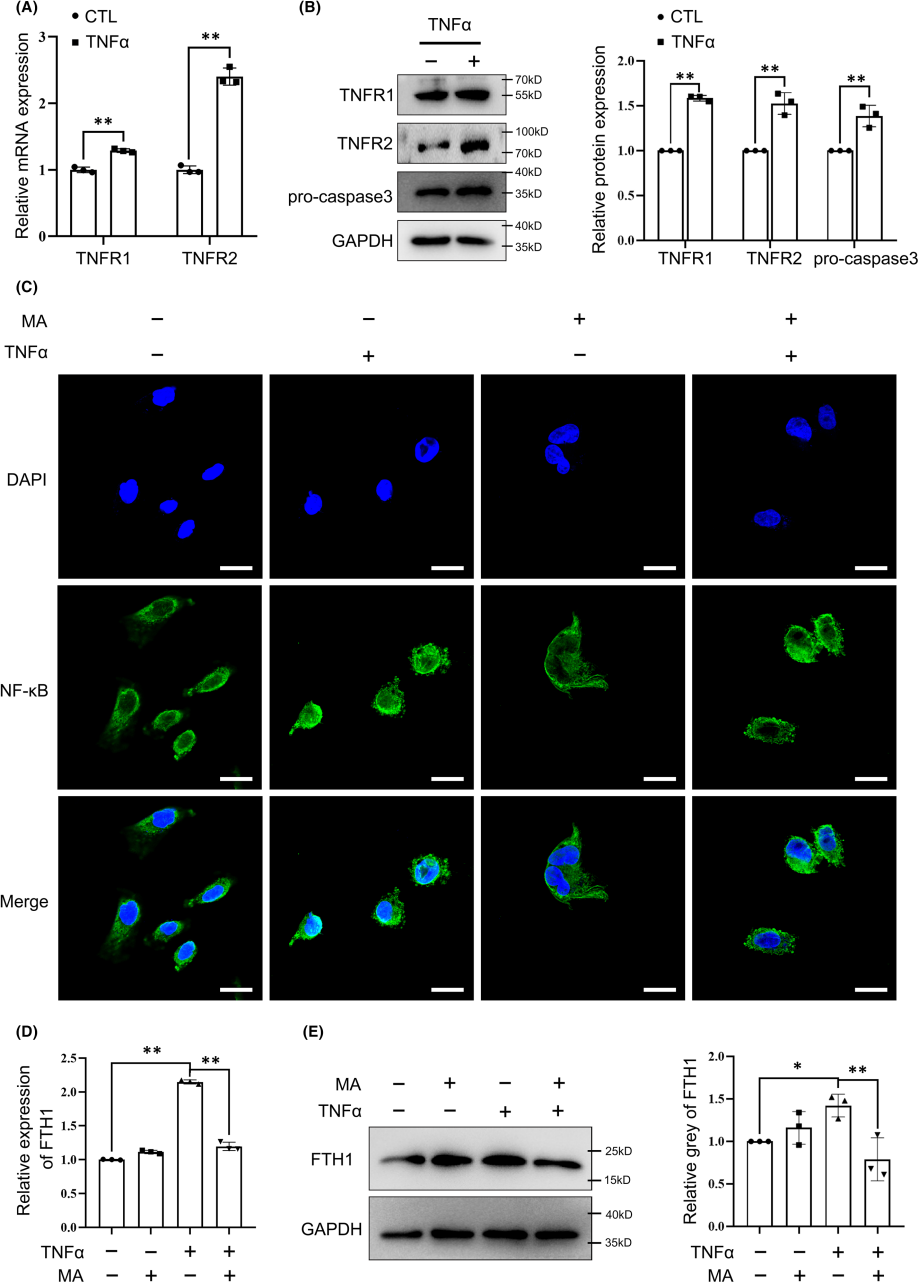
Expression of FTH1 was mediated by the axis of TNFα/TNFR/NF‐κB. A and B, RT‐PCR (A) and Western blotting (B) showed that treatment of 10 ng/ml TNFα for 48 h stimulated the elevated expression of TNFR1, TNFR2 and pro‐caspase3. C‐E, Stromal cells were treated with MA (20 μM) in the presence or absence of TNFα (10 ng/ml). The location of NF‐κB was observed by immunofluorescence staining (C). The expression of FTH1 was detected through RT‐PCR (D) and Western blotting (E). Scare bar = 20 μm. *p < 0.05, **p < 0.01

As literature reported, engagement of TNFα with its cognate receptors TNFR1 and TNFR2 results in the recruitment of many important adaptor proteins like TRADD, TRAF2, RIP and FADD, thereby activating and initiating downstream events leading to apoptosis, as well as NF‐κB activation, and so on.^30^, ^31^ To further understand the mechanism of TNFα‐induced apoptosis and the function of FTH1 in it, the downstream molecules were explored in the hESCs stimulated by TNFα. RT‐PCR and Western blotting showed that the expression of TNFR1 and TNFR2 was increased both at the transcription level and at the protein level (Figure 4A,B). In addition, NF‐κB was confirmed to translocate from the cytoplasm to the nucleus by immunofluorescence staining (Figure 4C), suggesting the activation of NF‐κB by TNFα. Furthermore, pro‐caspase3 was also induced by TNFα (Figure 4C), indicating the occurrence of apoptosis. The above results had demonstrated that FTH1 was upregulated by TNFα; thus, we speculated that FTH1 was the downstream target of NF‐κB through transcriptional regulation. JASPAR database (http://jaspar.genereg.net) was used to predict the transcription factor‐binding sites in the FTH1 promoter sequences,^32^ and NF‐κB was found to be likely to bind with the promoter of FTH1 (Table S2). To validate the binding, NF‐κB inhibitor maslinic acid (MA) was applied to repress the activation of NF‐κB. After being treated with MA, the nucleus translocation of NF‐κB was suppressed (Figure 4C); meanwhile, the increased expression of FTH1 was inhibited (Figure 4D,E). Together, the results support the notion that TNFα induces apoptosis through mediating of TNFR/NF‐κB/FTH1 axis in hESCs.

### 3.5 Validation of abnormal TNFR/NF‐κB/FTH1/apoptosis signals in human decidual tissues

**FIGURE 5:**
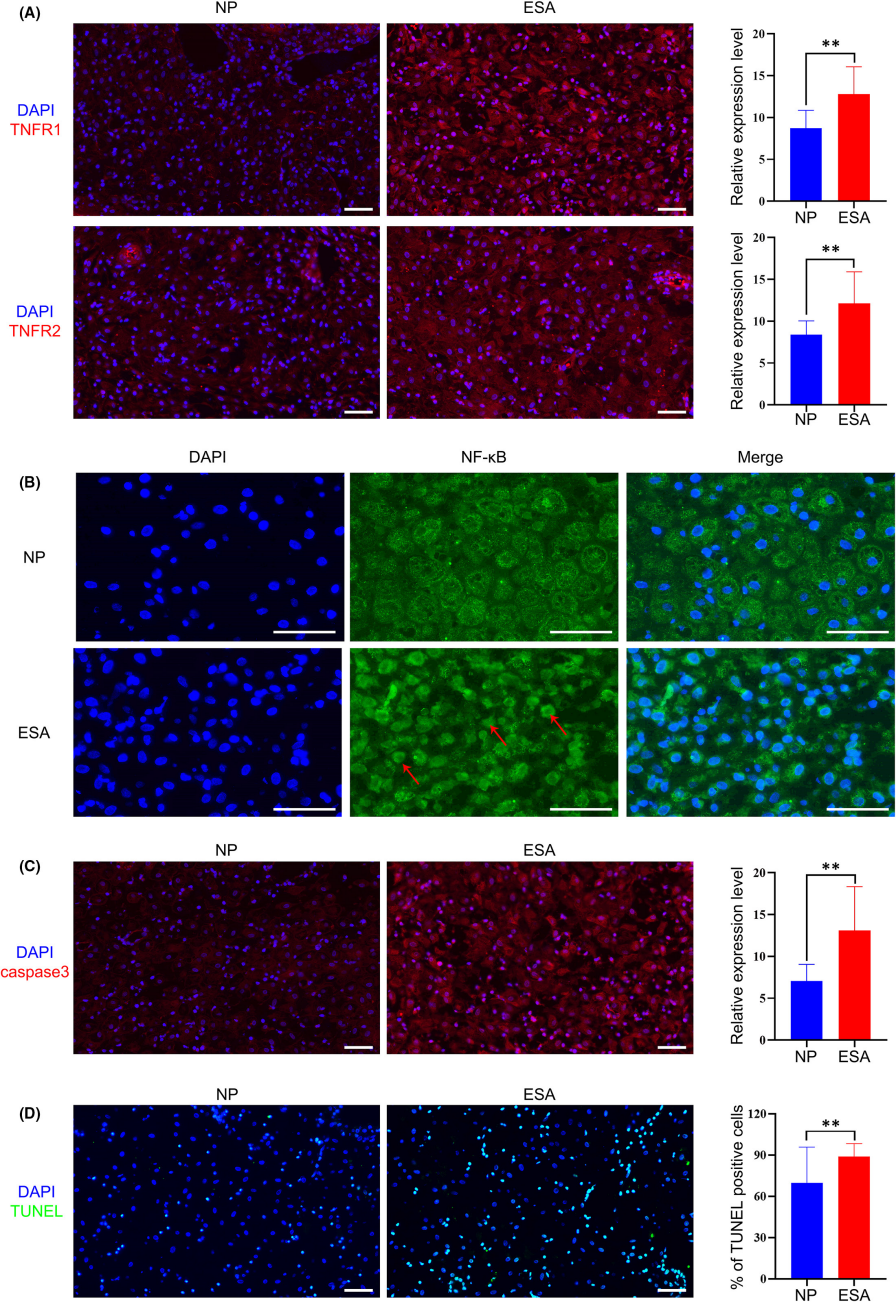
Validation of the activation of TNFR/NF‐κB/apoptosis signalling in the decidual stromal cells of the patient in the ESA group. (A) The fluorescence staining of the decidua from patients in NP group and ESA group showed that the expression of TNFR1 and TNFR2 was increased in the patient of ESA group. NP, *n* = 4; ESA, *n* = 4. **p < 0.01. Scare bar = 50 μm. (B) The NF‐κB signal was transactivated in the decidual stromal cells of the patient in the ESA group. Red arrow denotes the nuclear translocation of NF‐κB. NP, *n* = 3; ESA, *n* = 3. Scare bar = 50 μm. (C) Significantly increased staining with caspase3 in decidua in the ESA group compared to the NP group. NP, *n* = 4; ESA, *n* = 4. **p < 0.01. Scare bar = 50 μm. (D) The TUNEL staining showed significant elevation of apoptotic cells in the decidua from the ESA group. NP, *n* = 4; ESA, *n* = 4. **p < 0.01. Scare bar = 50 μm

**FIGURE 6:**
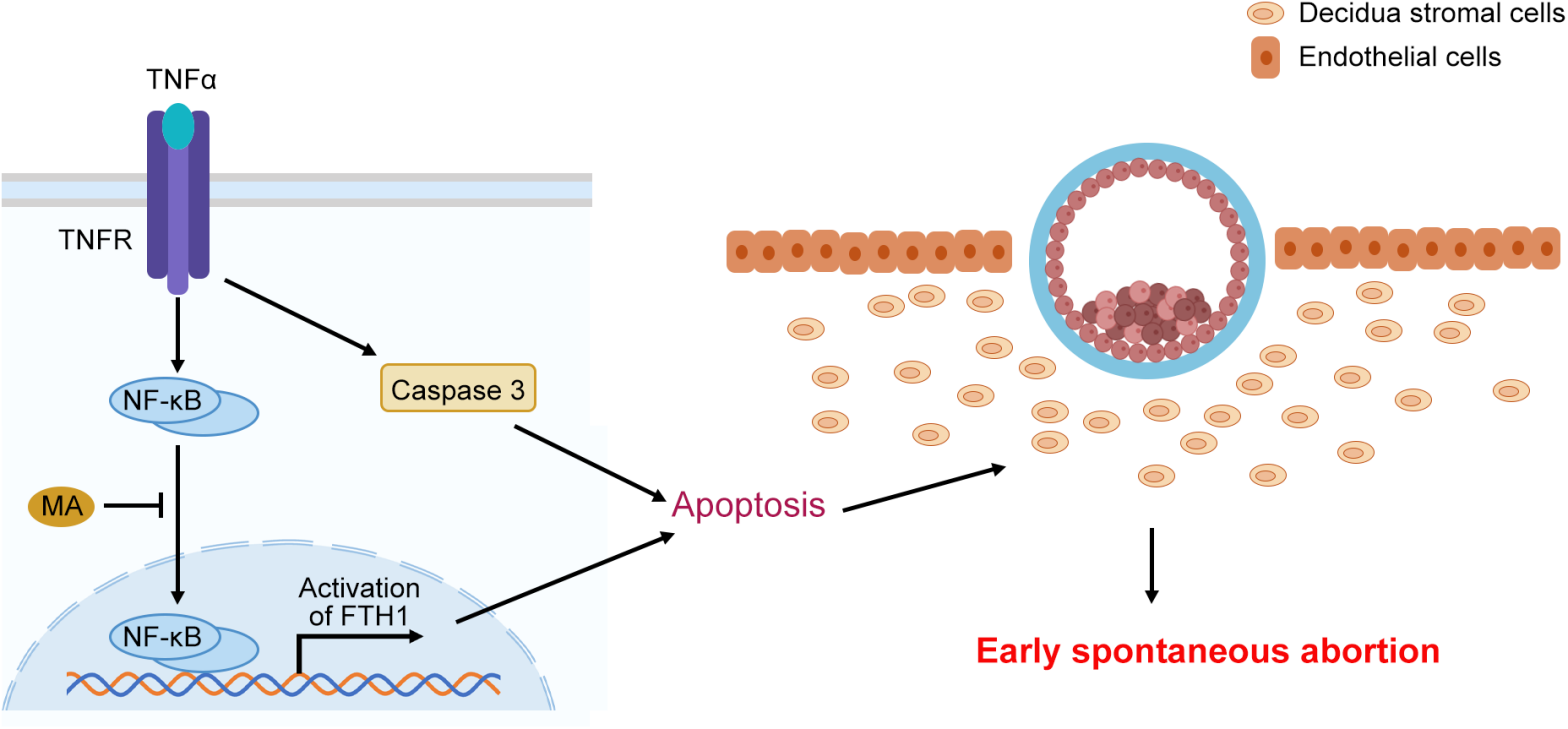
Hypothesis of the underlying pathogenesis for ESA induced by TNFα‐upregulated FTH1. The elevated TNFα induces the abnormally activated signals of TNFR/NF‐κB/FTH1 and leads to the excessive apoptosis of decidual stromal cells, which further results in unstable implantation of embryos and contributes to ESA

To elaborate that the occurrence of ESA was closely associated with the function of TNFα/TNFR/NF‐κB/FTH1/apoptosis signalling, the induction of this pathway was further testified in human decidual tissues. The immunofluorescence staining showed a significant enhancement of TNFR1 and TNFR2 in human decidual tissues from the ESA group (Figure 5A). Additionally, NF‐κB was nucleus translocated (Figure 5B), suggesting the activation of its transcriptional activity in the ESA group. Besides, decidual tissues from the ESA group exhibited a strongly positive staining of caspase3 (Figure 5C) and an obvious increase of TUNEL positive cells (Figure 5D), indicating the exacerbated apoptosis. These results were consistent with those data in hESCs and further verified the findings that the abnormal activation of TNFα/TNFR/NF‐κB/FTH1/apoptosis signalling may account for the pathogenesis of ESA, at least in some cases (Figure 6).

## 4 DISCUSSION

Previous evidences have emphasized the important role of ferritin in pregnancy; the imbalance of ferritin levels is often associated with adverse pregnancy outcomes.^21^, ^22^, ^24^, ^25^ Unfortunately, little researches focus on the functions of its subunits FTH1 during pregnancy. So far, just one literature has reported that FTH1 deletion would contribute to early embryonic lethality in mice,^16^ while whether the deficiency or excess of FTH1 would result in other pregnancy consequences remains to be explored. In the current study, we have demonstrated several novel points which could clarify the relationship between inflammation and FTH1 function in ESA pathogenesis, including (1) TNFα and FTH1 are upregulated in the decidual tissues from patients undergoing ESA; (2) the expression of FTH1 is mediated by TNFα/TNFR/NF‐κB axis; and (3) FTH1‐induced excessive apoptosis in response to TNFα signals may contribute to the development of ESA.

Many studies have claimed that ferritin especially FTH1 would be elevated together with aggravated infection or inflammation, which triggered by many pro‐inflammatory cytokines, in particular TNFα.^17^, ^33^, ^34^, ^35^ As early as 1988, Torti, et al. have revealed that the expression of FTH1 could be regulated by TNFα in mouse TA1 adipocytes and human muscle cells.^33^ Subsequently, similar conclusions are identified in lung macrophages,^34^ hepatocellular carcinoma cells,^35^ human periodontal ligament cells^17^ and so on. Intriguingly, we detected increased TNFα expression in local intrauterine decidual tissues from the ESA group, and discovered a similar upregulation of FTH1 which obviously positively correlated with the level of TNFα in our study. In subsequent experiments on hESCs, the expression of FTH1 was confirmed to be modulated by TNFα. Moreover, the expression of another subunit of ferritin FTL was also detected, although it was increased in decidual tissues from the ESA group and showed a strong correlation with TNFα, it was not modulated by TNFα in hESCs, implying the specific role of TNFα‐induced FTH1 in ESA.

To our knowledge, TNFα could activate apoptotic pathways via binding with TNFR1 containing the death domain, or enhance TNFα/TNFR1 mediated apoptotic signals by binding to TNFR2,^36^, ^37^ which may lead to embryo death, or unstable implantation of embryos, further contributing to ESA.^38^, ^39^ Meanwhile, the binding of TNFα to TNFR1 and TNFR2 induces the activation of NF‐κB as well, whereas the function of NF‐κB was controversial. Some researches suggest that NF‐κB could act as a blocker of apoptosis to protect the embryo from stimuli including TNFα, while some indicate that the activation of NF‐κB would aggravate apoptotic cell death through acting as a transcriptional factor to enhance the expression of target genes.^5^, ^36^ In the current study, we discovered the abnormal activation of TNFα‐induced TNFR/NF‐κB/FTH1/apoptosis signals both in hESCs and in decidual tissues from the ESA group, providing strong evidence that the dysregulated FTH1 signalling may be closely related with the occurrence of ESA. Nevertheless, long‐term treatment with TNFα could not induce the cell death of HTR8/SVneo, indicating that trophoblast cells may resistant to apoptosis signalling induced by TNFα.

Up to now, FTH1 is generally considered to have antioxidant properties that could protect cells from reactive oxygen species (ROS) due to its ferroxidase activity which could catalyse the conversion of Fe^2+^ into Fe^3+^.^17^, ^20^ Meanwhile, NF‐κB‐mediated FTH1 upregulation is thought to protect cells from apoptosis.^35^, ^40^ However, our results showed that TNFα could induce the apoptosis through activating the singals of NF‐κB/FTH1, which were opposite from the discovery reported by literatures. This may due to different diseases we focused on and different cell models we used, just like that the same concentration of TNFα could induce apoptosis in some cells, while showing a protective function in some cells.^35^, ^41^ Additionally, some researches have revealed the anti‐growth and apoptosis‐promoting effects of FTH1 on breast cancer cells and non‐small cell lung cancer cells, further illustrating the existence of inconsistency among different cells.^42^, ^43^

Decidua (a specialized endometrium) is an abundant source of cytokines, including TNFα, interleukin‐1α (IL‐1α), interleukin‐1β (IL‐1β), interleukin‐6 (IL‐6), interferon‐γ (IFN‐γ) and so on.^44^ The disorders of these cytokines would lead to adverse outcomes, containing miscarriage, preeclampsia, premature labour and so on.^45^, ^46^ However, the content of cytokines other than TNFα was not detected in our study, especially interleukin‐1β (IL‐1β), interleukin‐6 (IL‐6) and interferon‐γ (IFN‐γ), which are also considered risk stimuli of apoptosis and pregnancy loss.^7^, ^47^ Moreover, it has been reported that the FTH1 could be triggered by IL‐1α, IL‐1β, IL‐6 and IFN‐γ as well.^17^, ^48^, ^49^, ^50^ Therefore, it was difficult to conclude that the occurrence of ESA was just accounted for TNFα‐induced upregulation of FTH1. Since the literatures have proposed that FTH1 could be regarded as a marker to reflect iron stores of the body, and plays an important role in balancing iron homeostasis,^51^ it was suspectable that the increase of apoptosis may be related to the disorder of iron metabolism induced by dysregulation of FTH1. More studies are needed to understand the detailed mechanism underlying the association among inflammation, FTH1 and iron metabolism during pregnancy.

In conclusion, our study has found that the level of TNFα is significantly increased in the decidual tissues from the ESA group, and long‐term stimulation of high TNFα level is proved to induce the aberrant activation of TNFR/NF‐κB/FTH1 signals, which further led to the excessive apoptosis of the decidua, resulting in unstable implantation and ESA. This is the first study linking the TNFα‐upregulated FTH1 to the development of ESA, providing a possible target of drug therapy for ESA.

## CONFLICT OF INTEREST

The authors declare no conflict of interest.

## AUTHOR CONTRIBUTION

**Yuting Wen:** Formal analysis (equal); Validation (equal); Visualization (equal); Writing – original draft (equal). **Meng Cheng:** Resources (equal); Writing – review & editing (equal). **Lang Qin:** Supervision (equal); Writing – review & editing (equal). **Weinming xu:** Conceptualization (equal); Funding acquisition (equal); Supervision (equal); Writing – review & editing (equal).

## Supporting information

Figure S1

Figure S2

Figure S3

Table S1

Table S2

## Data Availability

Data are available on reasonable request. All data relevant of this study are available from the corresponding author on reasonable request.

## References

[1] Eroglu S , Colak E , Erinanc OH , et al. Serum and placental periostin levels in women with early pregnancy loss. J Reprod Immunol. 2020;140:103138. doi:10.1016/j.jri.2020.103138 32460058

[2] van den Berg MM , van Maarle MC , van Wely M , Goddijn M . Genetics of early miscarriage. Biochem Biophys Acta. 2012;1822(12):1951‐1959. doi:10.1016/j.bbadis.2012.07.001 22796359

[3] Clark DA , Coulam CB , Daya S , Chaouat G . Unexplained sporadic and recurrent miscarrage in the new millennium: a critical analysis of immune mechanisms and treatments. Hum Reprod Update. 2001;7(5):501‐511. doi:10.1093/humupd/7.5.501 11556498

[4] Jia CW , Wang L , Lan YL , et al. Aneuploidy in Early Miscarriage and its Related Factors. Chin Med J. 2015;128(20):2772‐2776. doi:10.4103/0366-6999.167352 26481744 PMC4736891

[5] Wang LQ , Yu XW , Yan CF , Wang X . Nuclear translocation of nuclear factor Kappa B in first trimester deciduas and chorionic villi in early spontaneous miscarriage women. Int J Mol Sci. 2010;11(2):521‐531. doi:10.3390/ijms11020521 20386652 PMC2852852

[6] Ticconi C , Pietropolli A , Di Simone N , Piccione E , Fazleabas A . Endometrial Immune Dysfunction in Recurrent Pregnancy Loss. Int J Mol Sci. 2019;20(21):5332. 10.3390/ijms20215332 10.3390/ijms20215332 PMC6862690 31717776

[7] Calleja‐Agius J , Jauniaux E , Pizzey AR , Muttukrishna S . Investigation of systemic inflammatory response in first trimester pregnancy failure. Hum Reprod (Oxford, England). 2012;27(2):349‐357. doi:10.1093/humrep/der402 22131390

[8] Griffith OW , Chavan AR , Protopapas S , Maziarz J , Romero R , Wagner GP . Embryo implantation evolved from an ancestral inflammatory attachment reaction. Proc Natl Acad Sci USA. 2017;114(32):E6566‐e6575. doi:10.1073/pnas.1701129114 28747528 PMC5559003

[9] Chavan AR , Griffith OW , Wagner GP . The inflammation paradox in the evolution of mammalian pregnancy: turning a foe into a friend. Curr Opin Genet Dev. 2017;47:24‐32. doi:10.1016/j.gde.2017.08.004 28850905

[10] Azizieh FY , Raghupathy RG . Tumor necrosis factor‐α and pregnancy complications: a prospective study. Med Princ Pract. 2015;24(2):165‐170. doi:10.1159/000369363 25501617 PMC5588217

[11] He Y , Sun Q . IFN‐γ induces upregulation of TNF‐α, downregulation of MMP‐2 and MMP‐9 expressions in abortion rat. Eur Rev Med Pharmacol Sci. 2018;22(15):4762‐4767. 10.26355/eurrev_201808_15609 30070307

[12] Zhang C , Deng X , Zhang X , et al. Association between Serum TNF‐α Levels and Recurrent Spontaneous Miscarriage: A Meta‐analysis. Am J Reprod Immunol. 2016;75(2):86‐93. 10.1111/aji.12447 26585408

[13] Li S , Wang L , Xing Z , Huang Y , Miao Z . Expression level of TNF‐α in decidual tissue and peripheral blood of patients with recurrent spontaneous abortion. Cent Euro J Immunol. 2017;42(2):156‐160. doi:10.5114/ceji.2017.69357 PMC5573888 28860933

[14] Casanova MJ , Chaparro M , Domènech E , et al. Safety of thiopurines and anti‐TNF‐α drugs during pregnancy in patients with inflammatory bowel disease. Am J Gastroenterol. 2013;108(3):433‐440. doi:10.1038/ajg.2012.430 23318480

[15] Johansen CB , Jimenez‐Solem E , Haerskjold A , Sand FL , Thomsen SF . The Use and Safety of TNF Inhibitors during Pregnancy in Women with Psoriasis: A Review. Int J Mol Sci. 2018;19(5):1349. doi:10.3390/ijms19051349 PMC5983707 29751529

[16] Ferreira C , Bucchini D , Martin ME , et al. Early embryonic lethality of H ferritin gene deletion in mice. J Biol Chem. 2000;275(5):3021‐3024. doi:10.1074/jbc.275.5.3021 10652280

[17] Huang W , Zhan Y , Zheng Y , Han Y , Hu W , Hou J . Up‐regulated ferritin in periodontitis promotes inflammatory cytokine expression in human periodontal ligament cells through transferrin receptor via ERK/P38 MAPK pathways. Clin Sci. 2019;133(1):135‐148. doi:10.1042/CS20180679 30552136

[18] McCullough K , Bolisetty S . Ferritins in Kidney Disease. Semin Nephrol. 2020;40(2):160‐172. doi:10.1016/j.semnephrol.2020.01.007 32303279 PMC7172005

[19] Goldfarb AN , Freeman KC , Sahu RK , et al. Iron control of erythroid microtubule cytoskeleton as a potential target in treatment of iron‐restricted anemia. Nat Commun. 2021;12(1):1645. 10.1038/s41467-021-21938-2 33712594 PMC7955080

[20] Fang X , Cai Z , Wang H , et al. Loss of Cardiac Ferritin H Facilitates Cardiomyopathy via Slc7a11‐Mediated Ferroptosis. Circ Res. 2020;127(4):486‐501. doi:10.1161/circresaha.120.316509 32349646

[21] Khambalia AZ , Collins CE , Roberts CL , et al. High maternal serum ferritin in early pregnancy and risk of spontaneous preterm birth. Br J Nutr. 2015;114(3):455‐461. doi:10.1017/s0007114515001932 26146276

[22] Xiao R , Sorensen TK , Frederick IO , et al. Maternal second‐trimester serum ferritin concentrations and subsequent risk of preterm delivery. Paediatr Perinat Epidemiol. 2002;16(4):297‐304. doi:10.1046/j.1365-3016.2002.00448.x 12445145

[23] Knovich MA , Storey JA , Coffman LG , Torti SV , Torti FM . Ferritin for the clinician. Blood Rev. 2009;23(3):95‐104. doi:10.1016/j.blre.2008.08.001 18835072 PMC2717717

[24] Guo Y , Zhang N , Zhang D , et al. Iron homeostasis in pregnancy and spontaneous abortion. Am J Hematol. 2019;94(2):184‐188. doi:10.1002/ajh.25341 30394565 PMC6687303

[25] Ramsey PS , Andrews WW , Goldenberg RL , Tamura T , Wenstrom KD , Johnston KE . Elevated amniotic fluid ferritin levels are associated with inflammation‐related pregnancy loss following mid‐trimester amniocentesis. J Matern Fetal Neonatal Med. 2002;11(5):302‐306. doi:10.1080/jmf.11.5.302.306 12389670

[26] Zhu LJ , Bagchi MK , Bagchi IC . Ferritin heavy chain is a progesterone‐inducible marker in the uterus during pregnancy. Endocrinology. 1995;136(9):4106‐4115. doi:10.1210/endo.136.9.7649119 7649119

[27] Livak KJ , Schmittgen TD . Analysis of relative gene expression data using real‐time quantitative PCR and the 2(‐Delta Delta C(T)) Method. Methods (San Diego, Calif). 2001;25(4):402‐408. doi:10.1006/meth.2001.1262 11846609

[28] Vermeulen K , Berneman ZN , Van Bockstaele DR . Cell cycle and apoptosis. Cell Prolif. 2003;36(3):165‐175. doi:10.1046/j.1365-2184.2003.00267.x 12814432 PMC6496173

[29] Whiteside EJ , Boucaut KJ , Teh A , Garcia‐Aragon J , Harvey MB , Herington AC . Elevated concentration of TNF‐alpha induces trophoblast differentiation in mouse blastocyst outgrowths. Cell Tissue Res. 2003;314(2):275‐280. doi:10.1007/s00441-003-0791-4 14505032

[30] Wajant H , Siegmund D . TNFR1 and TNFR2 in the Control of the Life and Death Balance of Macrophages. Front Cell Dev Biol. 2019;7:91. doi:10.3389/fcell.2019.00091 31192209 PMC6548990

[31] Chen G , Goeddel DV . TNF‐R1 signaling: a beautiful pathway. Science (New York, NY). 2002;296(5573):1634‐1635. doi:10.1126/science.1071924 12040173

[32] Fornes O , Castro‐Mondragon JA , Khan A , et al. JASPAR 2020: update of the open‐access database of transcription factor binding profiles. Nucleic Acids Res. 2020;48(D1):D87‐D92. doi:10.1093/nar/gkz1001 31701148 PMC7145627

[33] Torti SV , Kwak EL , Miller SC , et al. The molecular cloning and characterization of murine ferritin heavy chain, a tumor necrosis factor‐inducible gene. J Biol Chem. 1988;263(25):12638‐12644. 3410854

[34] Persson HL , Vainikka LK , Eriksson HB , Wennerström U . Lane‐Hamilton syndrome: ferritin protects lung macrophages against iron and oxidation. Chest. 2011;139(2):361‐367. doi:10.1378/chest.10-0818 20705801

[35] Kou X , Jing Y , Deng W , et al. Tumor necrosis factor‐α attenuates starvation‐induced apoptosis through upregulation of ferritin heavy chain in hepatocellular carcinoma cells. BMC Cancer. 2013;13(1):438. 10.1186/1471-2407-13-438 24066693 PMC3849379

[36] Toder V , Fein A , Carp H , Torchinsky A . TNF‐alpha in pregnancy loss and embryo maldevelopment: a mediator of detrimental stimuli or a protector of the fetoplacental unit? J Assist Reprod Genet. 2003;20(2):73‐81. doi:10.1023/a:1021740108284 12688591 PMC3455795

[37] Varfolomeev E , Vucic D . Intracellular regulation of TNF activity in health and disease. Cytokine. 2018;101:26‐32. doi:10.1016/j.cyto.2016.08.035 27623350

[38] Pampfer S . Dysregulation of the cytokine network in the uterus of the diabetic rat. Am J Reprod Immunol. 2001;45(6):375‐381. doi:10.1111/j.8755-8920.2001.450602.x 11458880

[39] Clark DA , Chaouat G , Gorczynski RM . Thinking outside the box: mechanisms of environmental selective pressures on the outcome of the materno‐fetal relationship. Am J Reprod Immunol. 2002;47(5):275‐282. doi:10.1034/j.1600-0897.2002.01093.x 12148542

[40] Pham CG , Bubici C , Zazzeroni F , et al. Ferritin heavy chain upregulation by NF‐kappaB inhibits TNFalpha‐induced apoptosis by suppressing reactive oxygen species. Cell. 2004;119(4):529‐542. doi:10.1016/j.cell.2004.10.017 15537542

[41] Zhang C , Tong T , Miao DC , Wang LF . Vitamin D inhibits TNF‐α induced apoptosis of human nucleus pulposus cells through regulation of NF‐kB signaling pathway. J Orthop Surg Res. 2021;16(1):411. 10.1186/s13018-021-02545-9 34183042 PMC8237490

[42] Biamonte F , Battaglia AM , Zolea F , et al. Ferritin heavy subunit enhances apoptosis of non‐small cell lung cancer cells through modulation of miR‐125b/p53 axis. Cell Death Dis. 2018;9(12):1174. 10.1038/s41419-018-1216-3 30518922 PMC6281584

[43] Ali A , Shafarin J , Abu Jabal R , et al. Ferritin heavy chain (FTH1) exerts significant antigrowth effects in breast cancer cells by inhibiting the expression of c‐MYC. FEBS Open Bio. 2021;11(11):3101‐3114. doi:10.1002/2211-5463.13303 PMC8564339 34551213

[44] Schatz F , Guzeloglu‐Kayisli O , Arlier S , Kayisli UA , Lockwood CJ . The role of decidual cells in uterine hemostasis, menstruation, inflammation, adverse pregnancy outcomes and abnormal uterine bleeding. Hum Reprod Update. 2016;22(4):497‐515. doi:10.1093/humupd/dmw004 26912000 PMC4917742

[45] Lash GE , Ernerudh J . Decidual cytokines and pregnancy complications: focus on spontaneous miscarriage. J Reprod Immunol. 2015;108:83‐89. doi:10.1016/j.jri.2015.02.003 25771398

[46] Erlebacher A . Immunology of the maternal‐fetal interface. Annu Rev Immunol. 2013;31:387‐411. doi:10.1146/annurev-immunol-032712-100003 23298207

[47] Haider S , Knöfler M . Human tumour necrosis factor: physiological and pathological roles in placenta and endometrium. Placenta. 2009;30(2):111‐123. doi:10.1016/j.placenta.2008.10.012 19027157 PMC2974215

[48] Festa L , Gutoskey CJ , Graziano A , Waterhouse BD , Meucci O . Induction of Interleukin‐1β by Human Immunodeficiency Virus‐1 Viral Proteins Leads to Increased Levels of Neuronal Ferritin Heavy Chain, Synaptic Injury, and Deficits in Flexible Attention. J Neurosc. 2015;35(29):10550‐10561. doi:10.1523/jneurosci.4403-14.2015 PMC4510293 26203149

[49] Gan ZS , Wang QQ , Li JH , Wang XL , Wang YZ , Du HH . Iron Reduces M1 Macrophage Polarization in RAW264.7 Macrophages Associated with Inhibition of STAT1. Mediators Inflamm. 2017;2017:1‐9. doi:10.1155/2017/8570818 PMC5327769 28286378

[50] Wei Y , Miller SC , Tsuji Y , Torti SV , Torti FM . Interleukin 1 induces ferritin heavy chain in human muscle cells. Biochem Biophys Res Comm. 1990;169(1):289‐296. doi:10.1016/0006-291x(90)91466-6 2350350

[51] Zhang N , Yu X , Xie J , Xu H . New Insights into the Role of Ferritin in Iron Homeostasis and Neurodegenerative Diseases. Mol Neurobiol. 2021;58(6):2812‐2823. doi:10.1007/s12035-020-02277-7 33507490

