# Supplementary figures and images for: TNFα‐induced abnormal activation of TNFR/NF‐κB/FTH1 in endometrium is involved in the pathogenesis of early spontaneous abortion
Source: J Cell Mol Med. 2022 Apr 20;26(10):2947–58. doi: 10.1111/jcmm.17308 (PMC9097845; doi:10.1111/jcmm.17308)

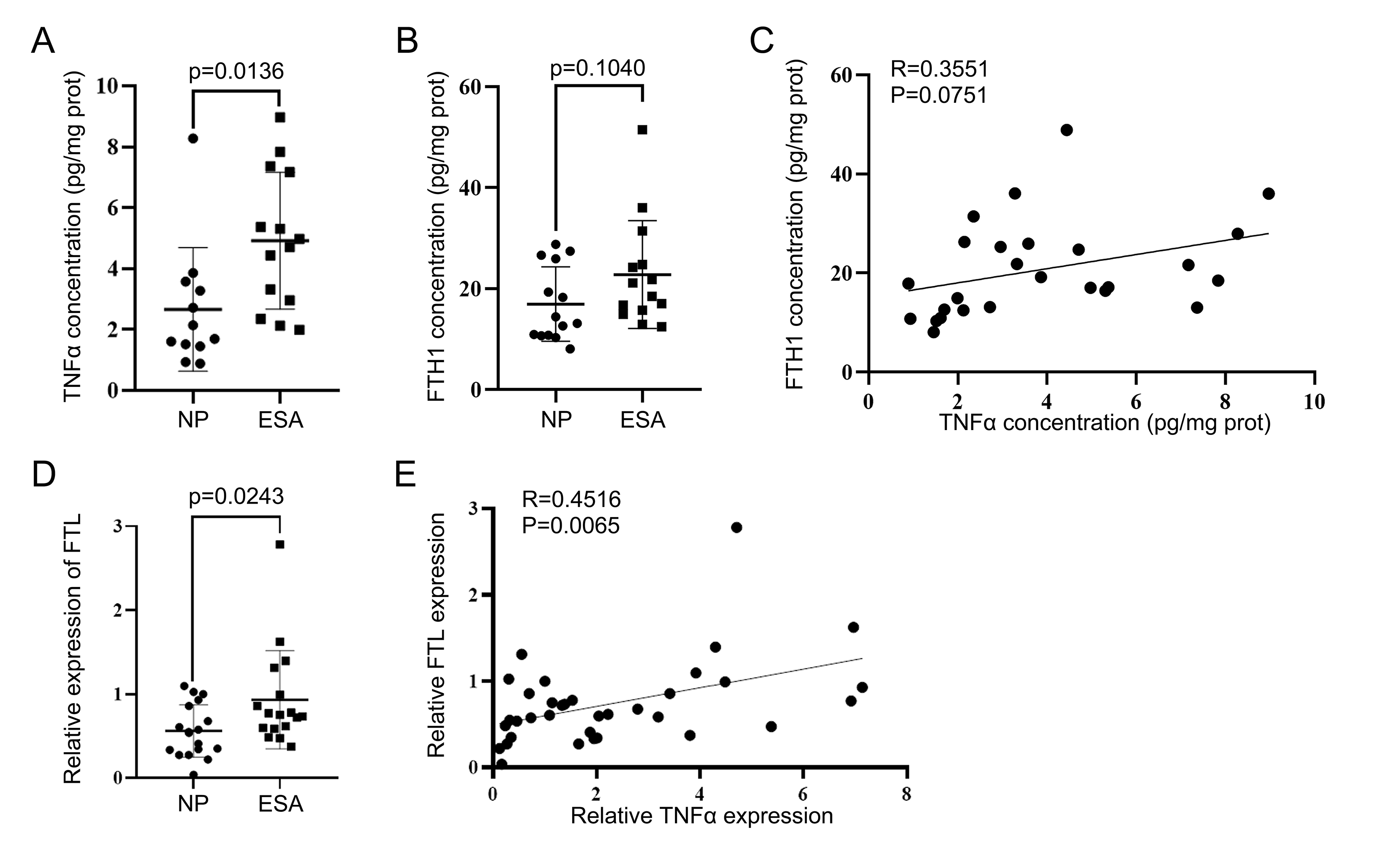

Supplement: Supplementary file 1 — Figure S1 [file JCMM-26-2947-s001.png]

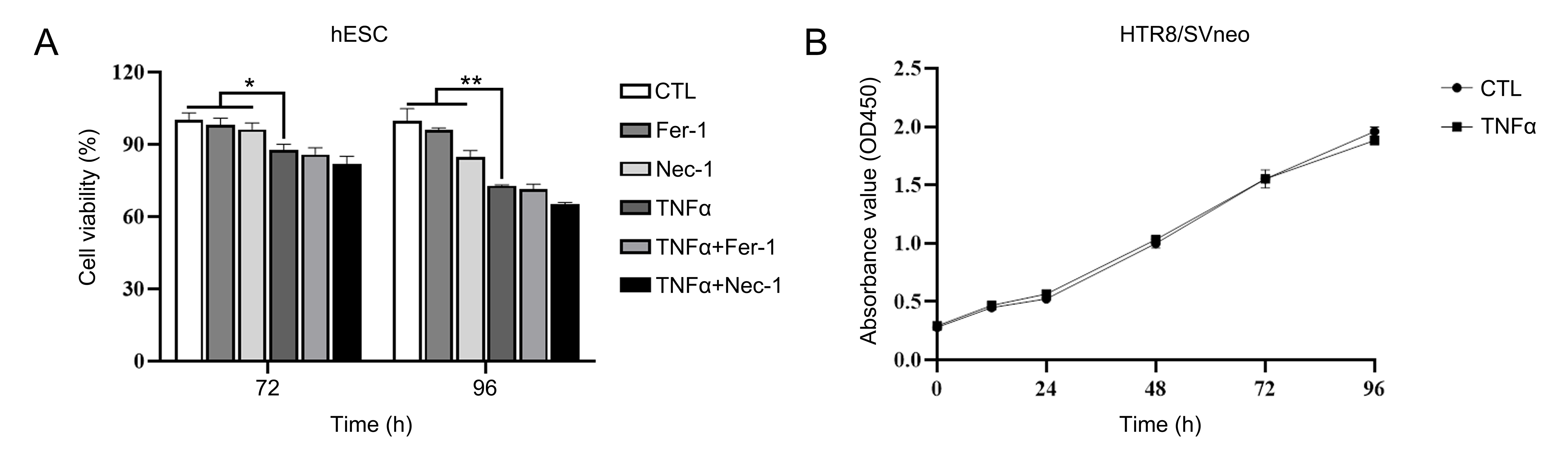

Supplement: Supplementary file 2 — Figure S2 [file JCMM-26-2947-s004.png]

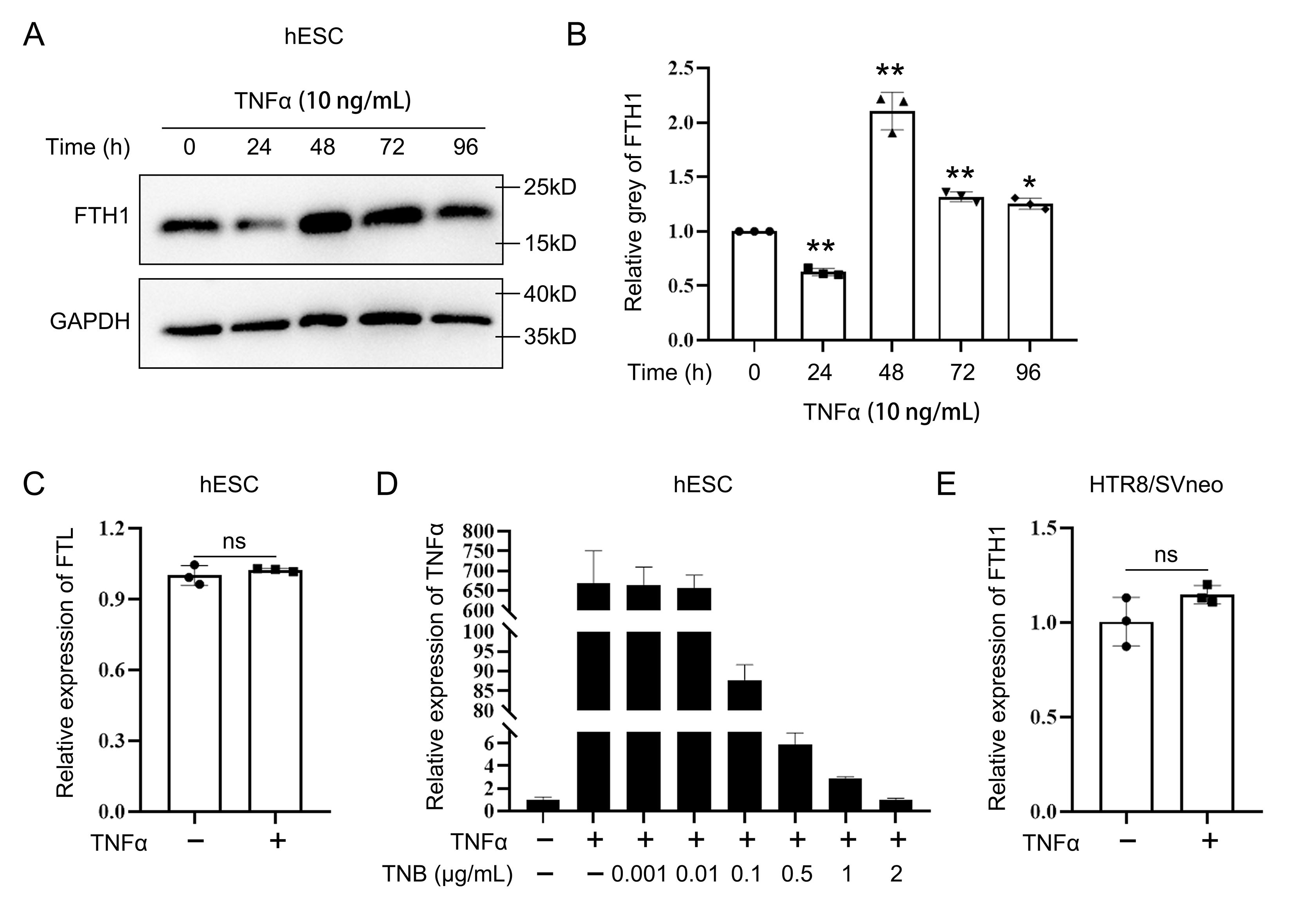

Supplement: Supplementary file 3 — Figure S3 [file JCMM-26-2947-s005.png]
