# Supplementary material for: TNFα‐induced abnormal activation of TNFR/NF‐κB/FTH1 in endometrium is involved in the pathogenesis of early spontaneous abortion
Source: J Cell Mol Med. 2022 Apr 20;26(10):2947–58. doi: 10.1111/jcmm.17308 (PMC9097845; doi:10.1111/jcmm.17308)
Supplement: Supplementary file 4 — Table S1 [file JCMM-26-2947-s002.docx]

**Supplementary Table 1 Primers used for human RT-PCR**

| Primer Name | Primer Sequence (5'-3') | Tm |
| --- | --- | --- |
| TNFα-F | GAACAATAGGCTGTTCCCATGTAG | 60℃ |
| TNFα-R | TGAATAGTAGGGCGATTACAGACA |  |
| TNFR1-F | CTATGCCCGAGTCTCAACCC | 60℃ |
| TNFR1-R | AGGTGAGGGACCAGTCCAAT |  |
| TNFR2-F | AAAGTAAGTACCACTCAGGCCAAC | 60℃ |
| TNFR2-R | GAGTAGAGAGATGGCTACGAGGAC |  |
| FTH1-F | GCTTCAACAGTGCTTGGACG | 60℃ |
| FTH1-R | GTCCTGGTGGTAGTTCTGGC |  |
| FTL-F | CCAGCACCGTTTTTGTGGTT | 60℃ |
| FTL-R | GCCAATTCGCGGAAGAAGTG |  |
| GAPDH-F | CTGGGCTACACTGAGCACC | 60℃ |
| GAPDH-R | AAGTGGTCGTTGAGGGCAATG |  |
