# Supplementary material for: TNFα‐induced abnormal activation of TNFR/NF‐κB/FTH1 in endometrium is involved in the pathogenesis of early spontaneous abortion
Source: J Cell Mol Med. 2022 Apr 20;26(10):2947–58. doi: 10.1111/jcmm.17308 (PMC9097845; doi:10.1111/jcmm.17308)
Supplement: Supplementary file 5 — Table S2 [file JCMM-26-2947-s003.docx]

**Supplementary Table 2 The binding sites of NF-κB on FTH1 promoter region obtained by the JASPAR database**

| Matrix ID | Name | Score | Relative score | Start | End | Strand | Predicted sequence |
| --- | --- | --- | --- | --- | --- | --- | --- |
| [9289](https://jaspar.uio.no/matrix/9289) | NFKB1 | 9.00639 | 0.85367 | 600 | 609 | - | GGGGAAGCCC |
| [9289](https://jaspar.uio.no/matrix/9289) | NFKB1 | 8.85329 | 0.84996 | 599 | 608 | + | AGGGCTTCCC |
| [9289](https://jaspar.uio.no/matrix/9289) | NFKB1 | 7.70076 | 0.82205 | 1309 | 1318 | - | CGGGTTTCCC |
| [9329](https://jaspar.uio.no/matrix/9329) | REL | 8.59622 | 0.86611 | 1310 | 1319 | - | CCGGGTTTCC |
| [9329](https://jaspar.uio.no/matrix/9329) | REL | 8.18192 | 0.85341 | 93 | 102 | + | TGTACTTTCC |
| [9329](https://jaspar.uio.no/matrix/9329) | REL | 7.61792 | 0.83611 | 1309 | 1318 | - | CGGGTTTCCC |
| [9329](https://jaspar.uio.no/matrix/9329) | REL | 7.53992 | 0.83372 | 1733 | 1742 | + | GTGGACTTCC |
| [9329](https://jaspar.uio.no/matrix/9329) | REL | 7.15859 | 0.82202 | 752 | 761 | - | GGGTGATTTC |
| [9329](https://jaspar.uio.no/matrix/9329) | REL | 6.64999 | 0.80643 | 1652 | 1661 | - | GGGGTGATCC |
| [9329](https://jaspar.uio.no/matrix/9329) | REL | 6.6469 | 0.80633 | 396 | 405 | + | TGGGATTACA |
| [9329](https://jaspar.uio.no/matrix/9329) | REL | 6.47253 | 0.80098 | 1153 | 1162 | - | AGGACTTTTC |
| [9333](https://jaspar.uio.no/matrix/9333) | NFKB1 | 10.7119 | 0.85866 | 599 | 609 | - | GGGGAAGCCCT |
| [9333](https://jaspar.uio.no/matrix/9333) | NFKB1 | 9.92874 | 0.84 | 599 | 609 | + | AGGGCTTCCCC |
| [9335](https://jaspar.uio.no/matrix/9335) | RELA | 8.5321 | 0.8237 | 1309 | 1318 | - | CGGGTTTCCC |
| [9335](https://jaspar.uio.no/matrix/9335) | RELA | 8.21726 | 0.81546 | 1310 | 1319 | - | CCGGGTTTCC |
| [9335](https://jaspar.uio.no/matrix/9335) | RELA | 8.08563 | 0.81201 | 93 | 102 | + | TGTACTTTCC |
| [10669](https://jaspar.uio.no/matrix/10669) | NFKB1 | 11.9722 | 0.92363 | 599 | 609 | + | AGGGCTTCCCC |
| [10669](https://jaspar.uio.no/matrix/10669) | NFKB1 | 9.4173 | 0.88636 | 93 | 103 | + | TGTACTTTCCA |
| [10669](https://jaspar.uio.no/matrix/10669) | NFKB1 | 9.37036 | 0.88567 | 1152 | 1162 | - | AGGACTTTTCA |
| [10669](https://jaspar.uio.no/matrix/10669) | NFKB1 | 9.25938 | 0.88405 | 1308 | 1318 | - | CGGGTTTCCCT |
| [10669](https://jaspar.uio.no/matrix/10669) | NFKB1 | 7.80753 | 0.86287 | 1565 | 1575 | + | AGGGCTTCTCC |
| [10669](https://jaspar.uio.no/matrix/10669) | NFKB1 | 5.67124 | 0.83171 | 1087 | 1097 | + | AGGGTTGTCCT |
| [10669](https://jaspar.uio.no/matrix/10669) | NFKB1 | 5.63966 | 0.83125 | 1734 | 1744 | + | TGGACTTCCTG |
| [10669](https://jaspar.uio.no/matrix/10669) | NFKB1 | 5.28749 | 0.82611 | 1168 | 1178 | + | TGGGCTTCGCC |
| [10669](https://jaspar.uio.no/matrix/10669) | NFKB1 | 3.70084 | 0.80296 | 750 | 760 | - | GGTGATTTCTT |
| [10669](https://jaspar.uio.no/matrix/10669) | NFKB1 | 3.68442 | 0.80272 | 1309 | 1319 | - | CCGGGTTTCCC |
| [10983](https://jaspar.uio.no/matrix/10983) | NFKB1 | 3.77332 | 0.80374 | 598 | 610 | - | CGGGGAAGCCCTC |
| [10984](https://jaspar.uio.no/matrix/10984) | NFKB2 | 8.47134 | 0.85283 | 598 | 610 | + | GAGGGCTTCCCCG |
| [10984](https://jaspar.uio.no/matrix/10984) | NFKB2 | 7.72934 | 0.84416 | 598 | 610 | - | CGGGGAAGCCCTC |
| [11354](https://jaspar.uio.no/matrix/11354) | RELB | 9.41399 | 0.86792 | 601 | 611 | + | GGCTTCCCCGA |
| [11354](https://jaspar.uio.no/matrix/11354) | RELB | 8.41604 | 0.84366 | 516 | 526 | + | AAATTCCCTTC |
| [11354](https://jaspar.uio.no/matrix/11354) | RELB | 8.25083 | 0.83964 | 75 | 85 | + | GCATCCCCCAA |
| [11354](https://jaspar.uio.no/matrix/11354) | RELB | 7.79005 | 0.82844 | 1278 | 1288 | + | CGACTCCCCGG |
| [11354](https://jaspar.uio.no/matrix/11354) | RELB | 7.00556 | 0.80937 | 229 | 239 | + | CGATTCTCCTG |
| [11354](https://jaspar.uio.no/matrix/11354) | RELB | 6.87376 | 0.80617 | 1675 | 1685 | + | CCCTTCCCCCG |
